# Cardioprotective effects of Prolame and SNAP are related with nitric oxide production and with diminution of caspases and calpain-1 activities in reperfused rat hearts

**DOI:** 10.7717/peerj.7348

**Published:** 2019-07-29

**Authors:** Nadia Giovanna Román-Anguiano, Francisco Correa, Agustina Cano-Martínez, Aurora de la Peña-Díaz, Cecilia Zazueta

**Affiliations:** 1 Departamento de Biomedicina Cardiovascular, Instituto Nacional de Cardiologia Ignacio Chávez, México, México; 2 Departamento de Fisiología, Instituto Nacional de Cardiologia Ignacio Chávez, México, México; 3 Departamento de Biología Molecular, Instituto Nacional de Cardiologia Ignacio Chávez, México, México; 4 Departamento de Farmacología, Universidad Nacional Autónoma de México, México, Mexico

**Keywords:** Cell death, Reperfusion, Caspases, Calpain, Nitric oxide, S-nitrosylation

## Abstract

Cardiac tissue undergoes changes during ischemia-reperfusion (I-R) that compromise its normal function. Cell death is one of the consequences of such damage, as well as diminution in nitric oxide (NO) content. This signaling molecule regulates the function of the cardiovascular system through dependent and independent effects of cyclic guanosine monophosphate (cGMP). The independent cGMP pathway involves post-translational modification of proteins by S-nitrosylation. Studies in vitro have shown that NO inhibits the activity of caspases and calpains through S-nitrosylation of a cysteine located in their catalytic site, so we propose to elucidate if the regulatory mechanisms of NO are related with changes in S-nitrosylation of cell death proteins in the ischemic-reperfused myocardium. We used two compounds that increase the levels of NO by different mechanisms: Prolame, an amino-estrogenic compound with antiplatelet and anticoagulant effects that induces the increase of NO levels in vivo by activating the endothelial nitric oxide synthase (eNOS) and that has not been tested as a potential inhibitor of apoptosis. On the other hand, S-Nitroso-*N*-acetylpenicillamine (SNAP), a synthetic NO donor that has been shown to decrease cell death after inducing hypoxia-reoxygenation in cell cultures. Main experimental groups were Control, I-R, I-R+Prolame and I-R+SNAP. Additional groups were used to evaluate the NO action pathways. Contractile function represented as heart rate and ventricular pressure was evaluated in a Langendorff system. Infarct size was measured with 2,3,5-triphenyltetrazolium chloride stain. NO content was determined indirectly by measuring nitrite levels with the Griess reaction and cGMP content was measured by Enzyme-Linked ImmunoSorbent Assay. DNA integrity was evaluated by DNA laddering visualized on an agarose gel and by Terminal deoxynucleotidyl transferase dUTP Nick-End Labeling assay. Activities of caspase-3, caspase-8, caspase-9 and calpain-1 were evaluated spectrophotometrically and the content of caspase-3 and calpain-1 by western blot. S-nitrosylation of caspase-3 and calpain-1 was evaluated by labeling S-nitrosylated cysteines. Our results show that both Prolame and SNAP increased NO content and improved functional recovery in post-ischemic hearts. cGMP-dependent and S-nitrosylation pathways were activated in both groups, but the cGMP-independent pathway was preferentially activated by SNAP, which induced higher levels of NO than Prolame. Although SNAP effectively diminished the activity of all the proteases, a correlative link between the activity of these proteases and S-nitrosylation was not fully established.

## Introduction

Nitric oxide (NO) is a short-life molecule produced after the oxidation of the guanidine group of L-arginine by NO synthases (NOS) (Moncada, Palmer & Higgs, 1991; Loscalzo & Welch, 1995). NO modulates cardiac function by regulating vascular tone, excitation-contraction coupling (Hammond & Balligand, 2012), platelet aggregability (Ikeda et al., 2000) and mitochondrial function (Zhao et al., 2005). Such effects are mainly associated either with the activation of soluble guanylate cyclase (sGC) that produces cyclic guanosine monophosphate (cGMP) and stimulates protein kinase G (PKG) or, with redox reversible modification of cysteine residues that results in the formation of S-nitrosothiols (SNO) in a process named S-nitrosylation (Stamler, Lamas & Fang, 2001). However, the finding that NO inhibits both Complex III and Complex IV activities in isolated mitochondria (Poderoso et al., 1996; Giuffrè et al., 1996) has raised the possibility of its role as a direct physiological regulator of mitochondrial respiration (Poderoso, Helfenberger & Poderoso, 2019).

It is well demonstrated that NO, either derived from NOS isoforms or exogenously administered protects against ischemia-reperfusion (I-R)-induced injury (Bolli, 2001; Jones et al., 2004; Hu et al., 2016); however, the preponderance of cGMP-dependent or of cGMP-independent pathways in such protection is still under debate. It was described for example, that sGC inhibition abrogate the post-conditioning infarct-sparing effect in rabbit hearts, suggesting a preponderant role of cGMP-dependent mechanisms (Yang et al., 2004, 2005). On the other hand, we and others have reported that NO-mediated cardioprotection might be also regulated by S-nitrosylation signaling. Sun et al. (2013) demonstrated that blockage of the sGC/cGMP/PKG signaling pathway did not affect ischemic postconditioning-mediated cardioprotection and this finding was correlated with increased SNO levels; whereas our group described the partial recovery of heart function when the NO donor: (*Z*)-1-[*N*-(2-aminoethyl)-*N*-(2-ammonioethyl)amino]diazen-1-ium-1,2-diolate (DETA-NO) was administrated to postconditioned hearts in which sGC was inhibited (Correa et al., 2015).

NO-mediated cardioprotection has been associated with S-nitrosylation of diverse proteins, like intracellular calcium handling proteins (e.g., L-type Ca^2+^ channels and sarcoplasmic reticulum calcium pump SERCA2a) (Sun et al., 2007), mitochondrial proteins (Sun et al., 2015) and antioxidant response proteins (Tao et al., 2004), but it has not been demonstrated if the activity of cell death-related proteins are under S-nitrosylation regulation in reperfusion damage. Also, as increased SNO may have potential clinical significance, it is relevant to determine if NO produced by the canonical protein kinase B (Akt)/endothelial nitric oxide synthase (eNOS) signaling or that produced by NO donors induce different levels of S-nitrosylation in the context of cardioprotection.

Therefore, the goal of this study was to determine if proteins related with cell death are susceptible to S-nitrosylation regulation in isolated hearts treated either with the estrogen analog Prolame, that increases NO levels through the activation of the PI3K/Akt/eNOS signaling pathway (Hernández-Reséndiz et al., 2015) and with the synthetic NO donor S-Nitroso-*N*-acetylpenicillamine (SNAP) which bypasses eNOS activity.

## Material and Methods

### Reagents

Chemicals were of reagent or higher grade from Sigma-Aldrich (St Louis, MO, USA) unless otherwise specified. Anti-caspase-3 monoclonal (sc-136219), anti-calpain-1 monoclonal (sc-271313), agarose-conjugated (AC) anti-caspase-3 (sc-136219) and AC anti-calpain-1 antibodies (sc-271313) were from Santa Cruz Biotechnology (Santa Cruz, CA, USA). Fluorogenic calpain-1 substrate (208748) and colorimetric caspase-3 substrate (235400) were from Calbiochem (Darmstadt, Germany); colorimetric caspase-8 substrate (260-045-M005) and colorimetric caspase-9 substrate (260-081-M005) were from Enzo Life Sciences (Farmingdale, NY, USA). The enhanced chemiluminescence detection system was from Millipore Corporation (Bedford, MA, USA) and horseradish peroxidase-conjugated secondary antibodies were from Santa Cruz Biotechnology (Santa Cruz, CA, USA); In situ Cell Death Detection Kit, Fluorescein (11-684-795-910) was from Roche; Cyclic GMP Enzyme-linked Immunosorbent Assay Kit was purchased from Cayman Chemical (Ann Arbor, MI, USA); whereas Pierce S-Nitrosylation Western Blot Kit was from Thermo Scientific. 1H-(1,2,4)Oxadiazolo(4,3-a)quinoxalin-1-one (ODQ) was from Calbiochem (Darmstadt, Germany) and 17β-(3-hydroxy-1-propylamino)-1,3,5(10)-estratrien-3-ol, Prolame, was synthesized and chemical purity established as previously reported (Fernández-G et al., 1985).

### Ethical approval

The investigation was approved by the Ethics Committee of the National Institute of Cardiology, “Ignacio Chávez” (14CB09012016) and the experimental protocols followed the guidelines of Norma Oficial Mexicana for the use and care of laboratory animals (NOM-062-ZOO-1999) and for disposal of biological residues (NOM-087-SEMARNAT-SSA1-2002).

### Experimental design

**Figure 1:**
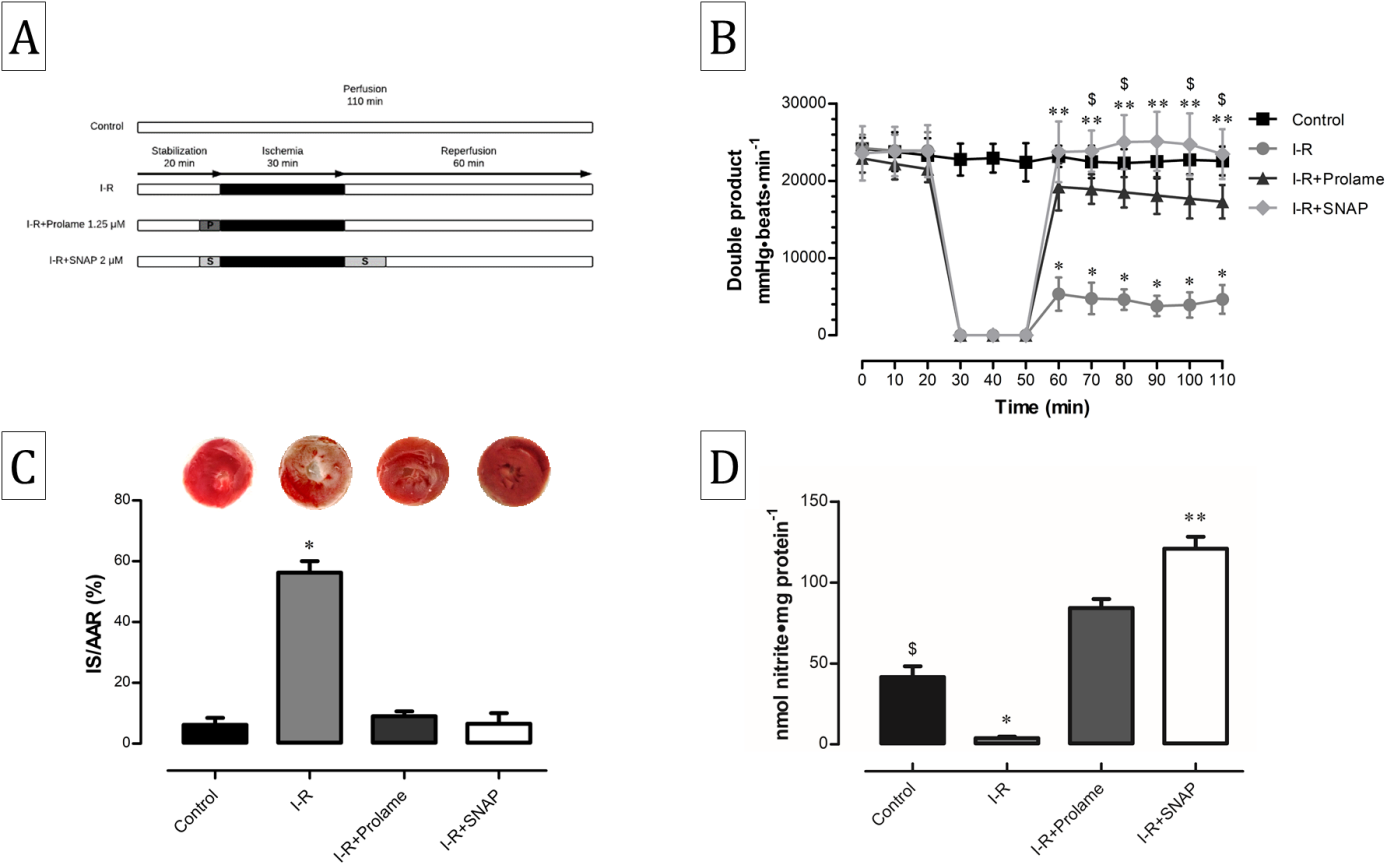
Cardiac function, infarct size and nitrite content in reperfused hearts treated with Prolame or SNAP. (A) Schematic representation of the experimental procedures performed in the Langendorff model. Control group was perfused during 110 min; ischemia-reperfusion (I-R) group, with 30 min of ischemia and 60 min of reperfusion; I-R+Prolame group, in which Prolame (1.25 μm) was added only 5 min before ischemia; I-R+SNAP group, in which SNAP (two μm) was added 5 min before ischemia and 10 min at the start of reperfusion. (B) Cardiac function expressed as the double product (DP = LVDP × heart rate) of the experimental groups. Values are expressed as mean ± SD of eight independent heart preparations per group. The statistical test used was two-way ANOVA. ^∗^*P* < 0.001 I-R vs. all groups; ^∗∗^*P* < 0.001 I-R+SNAP vs. I-R+Prolame; ^$^*P* < 0.05 Control vs. I-R+Prolame. (C) Representative images of TTC-stained hearts and infarct size quantification expressed as percentage of infarct size/area at risk. Values are expressed as mean ± SD of five independent heart preparations per group. The statistical test used was one-way ANOVA. ^∗^*P* < 0.05 I-R vs. all groups. (D) Nitric oxide content in the experimental groups. Values are expressed as mean ± SD of three independent heart preparations per group. The statistical test used was one-way ANOVA. ^∗^*P* < 0.05 I-R vs. all groups; ^∗∗^*P* < 0.05 I-R+SNAP vs. I-R+Prolame; ^$^*P* < 0.05 Control vs. I-R+Prolame and I-R+SNAP.

Male wistar rats (300–350 g) were anaesthetized by injecting intraperitoneally a single dose of sodium pentobarbital (60 mg/kg i.p.) plus sodium heparin and complete lack of pain response was assessed by determining pedal withdrawal reflex. Hearts were perfused retrogradely on a Radnoti Langendorff heart perfusion system (ADInstruments, Sydney, NSW, Australia) via the aorta at a constant flow rate of 12 ml/min with Krebs-Henseleit solution (118 mM NaCl, 4.75 mM KCl, 1.18 mM KH_2_PO_4_, 1.18 mM MgSO_4_ · 7H_2_O, 2.5 mM CaCl_2_, 25 mM NaHCO_3_, five mM glucose and 0.1 mM sodium octanoate, pH 7.4), which was continuously bubbled with 95% O_2_ and 5% CO_2_ at 37 °C. Cardiac performance was measured at left ventricular end-diastolic pressure of 10 mmHg using a latex balloon inserted into the left ventricle and connected to a pressure transducer that, in turn, was connected to a PowerLab System (ADInstruments, Sydney, NSW, Australia). Throughout the experiment, left ventricular developed pressure (LVDP) and heart rate (HR) was calculated automatically from the pressure trace with the digital acquisition system LabChart 8.1.5 Pro (ADInstruments, Sydney, NSW, Australia). HR was expressed as beat number min^−1^ and the double product (DP) was calculated by multiplying HR by LVDP. Hearts were perfused for 20 min to reach a steady state and then subjected to the different protocols. The experimental groups were: (1) Control, hearts perfused for additional 110 min; (2) I-R, hearts subjected to global ischemia for 30 min by turning off the pumping system and 60 min of reperfusion; (3) I-R+Prolame, hearts that received 1.25 μM of the compound for 5 min before ischemia; (4) I-R+SNAP, hearts perfused with two μM of the compound during 5 min before ischemia and 10 min during reperfusion (Fig. 1A). These concentrations were chosen after performing dose-response experiments of heart function recovery.

Additional experiments were performed administrating 15 μM L-NAME, inhibitor of the eNOS and 50 μM ODQ, inhibitor of the sGC before the addition of Prolame and SNAP, as previously reported (Correa et al., 2015). Both SNAP and ODQ are light sensitive, therefore heart perfusion and sample preparation were performed in the dark. At the end of the experiments, unless otherwise indicated, heart tissue was preserved in cold buffer (100 mM Tris–HCl, 145 mM NaCl, 10 mM EDTA, pH 7.3).

### Infarct size measurement

Infarct size was measured using 2,3,5-triphenyltetrazolium chloride (TTC) staining (Ramírez-Camacho et al., 2016). Heart was frozen and cut into three mm transverse slices. The slices were incubated in 1% TTC solution for 20 min at 37 °C and then immersed in formalin solution for 20 min to enhance the contrast between stained and unstained tissue. TTC stains living tissue into a deep red color. Digital images of heart slices were obtained and analyzed using the ImageJ 1.48 software (NIH, Bethesda, MD, USA). Infarct size was expressed as a percentage and calculated by dividing the area of infarct by total area at risk.

### Preparation of tissue homogenates

Cardiac fresh tissue was frozen in liquid nitrogen, pulverized with a pistil in a mortar and homogenized in a buffer containing 100 mM HEPES, 20% glycerol and 0.5 mM EDTA, pH 7.5 and centrifuged at 10,000×*g* for 10 min at 4 °C. The supernatant fraction was recovered and immediately frozen in liquid nitrogen. Protein was measured by the Lowry method (Lowry et al., 1951).

### Preparation of cytosolic fractions

Cardiac fresh tissue was minced with scissors and homogenized in Tris-buffered saline (100 mM Tris–HCl, 145 mM NaCl, pH 7.3) plus EDTA (10 mM) and centrifuged at 10,000×*g* for 10 min at 4 °C. The cytosolic fraction was recovered after centrifuging the supernatant at 100,000×*g* for 60 min and immediately frozen in liquid nitrogen (Kohli et al., 1997). Protein was measured by the Lowry method (Lowry et al., 1951).

### Determination of nitrite levels

Nitrite levels were measured using the colorimetric Griess reagent as indicator of NO production. Fresh cardiac tissue was homogenized in 50 mM Tris, 120 mM NaCl, 0.05% IGEPAL, pH 8.0, centrifuged at 10,000×*g* for 20 min at 4 °C and the supernatants filtrated through syringe-driven filter units (Merck KGaA, Darmstadt, Germany) to eliminate proteins from each sample. Nitrates were reduced to nitrites with bacterial nitrate reductase of *E. Coli*. The samples were then incubated with 0.2 ml of Griess solution (0.1% sulfanilamide and 0.5% of N-(1-naphthyl)ethylenediamine dihydrochloride in phosphoric acid 2.5%) for 60 min at 37 °C in the dark. The formed azo dye was spectrophotometrically quantified at 543 nm and compared against a sodium nitrate concentration curve incubated with the enzyme.

### cGMP content

Cyclic guanosine monophosphate levels were measured by competitive Cyclic GMP EIA kit (Cayman Chemical, Ann Arbor, MI, USA) in homogenates from the indicated experimental groups according to the manufacturer’s instructions.

### DNA Fragmentation assay

Frozen cardiac tissue (five mg) was pulverized and incubated with 0.5 ml extraction buffer (10 mM Tris, 100 mM EDTA, 5% SDS; pH 8.0) plus 20 μg/ml of RNAse for 1 h at room temperature. Then, 200 μg/ml of Proteinase K were added and incubated overnight at 50 °C. An equal volume of phenol adjusted with Tris buffer was added to the samples and maintained under constant agitation during 60 min at 4 °C. The samples were centrifuged at 5,000×*g* for 30 min at room temperature; the aqueous-viscous phase was carefully transferred to a new tube and the extraction was repeated with an equal volume of phenol/chloroform (1:1 v/v). The aqueous phase was transferred to a new tube and the DNA precipitated by adding 0.1 volumes of 3M sodium acetate and two volumes of absolute ethanol. The samples were centrifuged at 5,000×*g* for 20 min at room temperature; the supernatant was carefully removed, and 0.5 ml of 70% ethanol was added to rinse the DNA, which was dissolved in extraction buffer. DNA was analyzed in a 1.5% agarose gel with 0.5 μg/ml ethidium bromide.

### TUNEL assay

Samples of ventricular tissue were fixed in 4% paraformaldehyde, washed with PBS three times for 5 min at room temperature and incubated in 30% sucrose for 24 h at 4 °C. Transversal cryosections of 10 μm from each heart were obtained (Minotome PLUS™, Digital Microtome Cryostat, Triangle Biomedical Sciences (TBS, Inc.), USA) and mounted on gelatinized slides. The tissue sections were rehydrated in PBS for 30 min at room temperature, permeabilized for 2 min at 4 °C with a fresh solution of 0.1% triton X-100 and 0.1% sodium citrate and washed with PBS two times for 5 min at room temperature. Each tissue section was rounded with hydrophobic pen and incubated with Terminal deoxynucleotidyl transferase dUTP Nick-End Labeling (TUNEL) reaction mixture (terminal deoxy nucleotide transferase plus nucleotide mixture in reaction buffer) in a humidified atmosphere at 37 °C in the dark for 60 min. After incubation, the slides were washed with PBS three times for 5 min at room temperature, counterstained with 4′,6-Diamidino-2-phenylindole dihydrochloride to visualize the nucleus and mounted for fluorescence microscopy analysis (Floid Cell Imaging Station; Life Technologies, Carlsbad, CA, USA). Tissue sections of negative (without transferase in the buffer reaction) and positive controls (pretreated with 3,000 U/ml recombinant DNase I in Tris–HCl 50 mM, pH 7.5, one mg/ml BSA) were included in the assay.

### Caspase-3, -8 and -9 activity

Caspase-3, -8 and -9 activities were measured using 200 μM of the colorimetric substrate Ac-DEVD-pNA, Ac-IETD-pNA and Ac-LEHD-pNA respectively, in a total volume of 0.3 ml, containing 30 μl of the cytosolic fraction and 240 μl of HEPES buffer (100 mM HEPES, 20% glycerol, five mM DTT, 0.5 mM EDTA, pH 7.5) at 37 °C for 60 min in 96-microwell plates. Changes in absorbance were evaluated at 405 nm in a microplate reader (BioTek Instruments, Inc., Winooski, VT, USA). The activities of caspases were normalized by mg of protein.

### Calpain-1 activity

Fifty μg of cytosolic fractions, 10 mM CaCl_2_ and 10 μM of the synthetic fluorogenic substrate for calpain H-K (FAM)-EVY∼GMMK (DABCYL)-OH (Merck Darmstadt, Germany) were added to reaction buffer containing 100 mM Tris–HCl, 145 mM NaCl, pH 7.3 in a total volume of 0.2 ml and incubated at 37 °C for 60 min in 96-microwell plates. To measure calcium-independent activity, CaCl_2_ was replaced with reaction buffer. Increase in fluorescence was measured in a microplate reader (BioTek Instruments, Inc., Winooski, VT, USA) at λ_em_ and λ_ex_ of 518 and 490 nm, respectively. The calpain activity was normalized by mg of protein.

### Immunoprecipitation of caspase-3 and calpain-1

Frozen cardiac tissue from the different groups were homogenized in PBS buffer (136.9 mM NaCl, 2.67 mM KCl, 8.1 mM Na_2_HPO_4_, 1.47 mM KH_2_PO_4_; pH 7.4). The homogenates were maintained under constant agitation for 2 h at 4 °C and then centrifuged at 10,000×*g* for 20 min at 4 °C. The supernatants were recovered, placed in a new tube in cold and protein was measured by the Lowry method. Duplicated samples containing the same amount of protein (one-mg) were taken to a final volume of one ml with PBS buffer. Then, five μl of calpain-1 antibody or of caspase-3 antibodies coupled to agarose (slurry) were added to each sample and incubated overnight at 4 °C under constant agitation. The samples were centrifuged at 3,000×*g* for 2 min at 4 °C, the supernatant was carefully removed, and one ml of PBS was added and gently inverted by hand to wash the pellet. This step was repeated one more time and the pellet was recovered to evaluate total protein and S-nitrosylation levels.

### S-nitrosylation assay

Protein S-nitrosylation was measured by selective reduction and labeling of S-nitrosylated cysteines of the immunoprecipitated proteins with the Pierce S-Nitrosylation Western Blot Kit (Thermo Scientific, Waltham, MA, USA) according to the manufacturer’s instructions. As the cysteine biotinylation is reversible, the samples were prepared without reducing agents.

### Statistical analysis

Data analysis was performed by analysis of variance followed by Bonferroni’s test, using the Graph Pad PRISM 5 for windows version 5.03 software. Results were expressed as mean ± SD or as otherwise indicated; *P*-values < 0.05 were considered statistically significant.

## Results

### Prolame and SNAP maintain cardiac function and reduce infarct size in reperfused hearts

Cardiac function expressed as the DP was maintained in the Control group during 110 min of constant reperfusion. DP decreased early and until the end of reperfusion in the I-R group (*P* < 0.05). Both SNAP and Prolame groups recovered cardiac function during reperfusion (Fig. 1B). Accordingly, infarct size decreased in hearts treated with Prolame or SNAP compared with the group I-R (9 ± 1.6 and 6.5 ± 3.5 vs. 56.2 ± 3.7; respectively; Fig. 1C).

### Prolame preserves and SNAP increases nitric oxide in I-R hearts

Nitrite content, an indicator of NO levels diminished significantly in I-R homogenates as compared with the Control group (3.8 ± 0.9 vs. 41.5 ± 6.7 nmol nitrite/mg protein; *P* < 0.05). Conversely, both Prolame and SNAP increased the levels of nitrite even to higher levels than those measured in the Control group (84.1 ± 5.6 and 120.8 ± 7.3 nmol nitrite/mg protein, respectively; *P* < 0.05, Fig. 1D). These data correlate with heart function recovery and decrease of cell death.

### Contribution of cGMP-dependent and independent nitric oxide pathways in the cardioprotective effect of Prolame and SNAP

**Figure 2:**
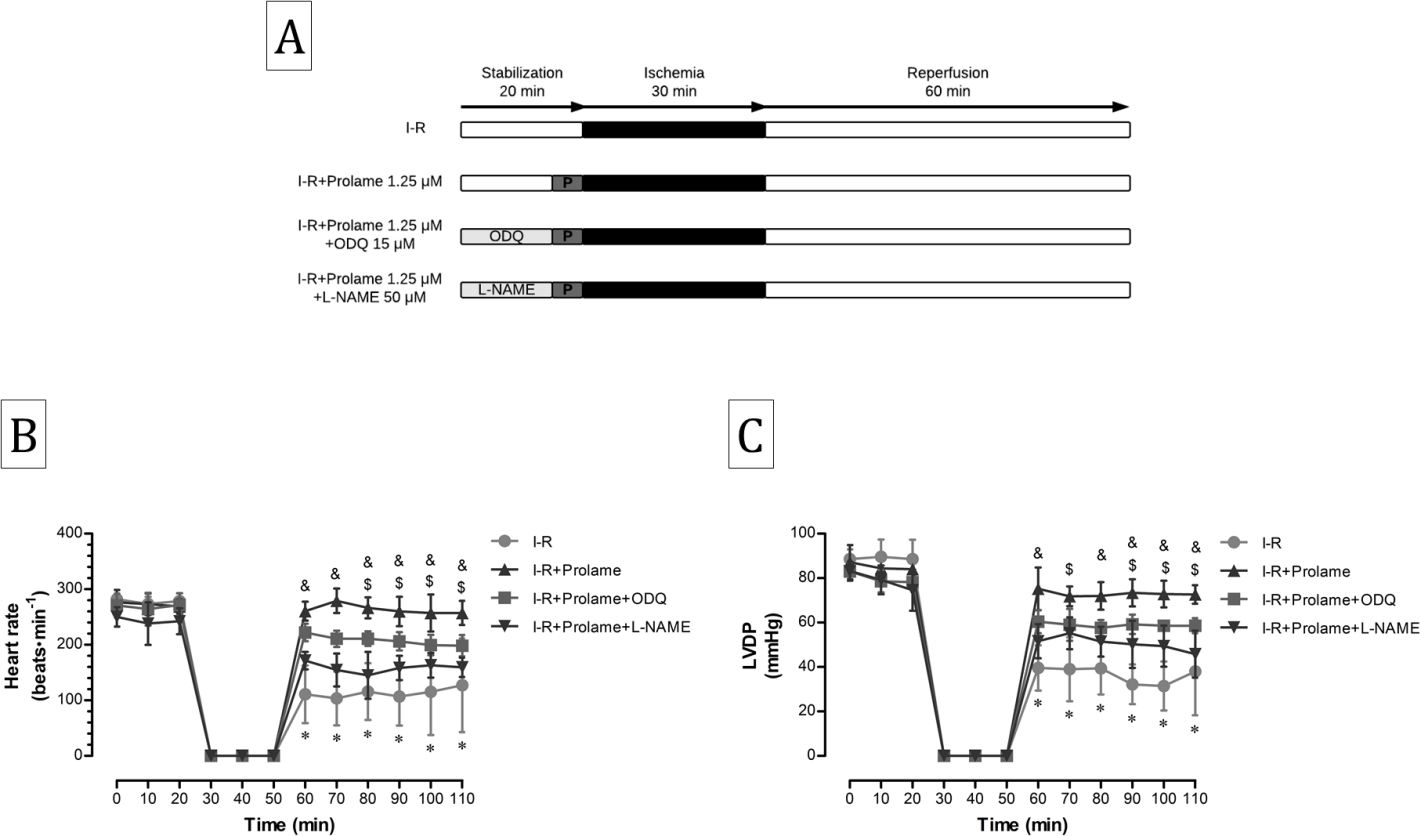
Effect of ODQ and L-NAME on the cardioprotection conferred by Prolame. (A) Schematic representation of the experimental procedure in the Langendorff model using the inhibitory compounds ODQ and L-NAME. Ischemia-reperfusion (I-R) group, with 30 min of ischemia and 60 min of reperfusion; I-R+Prolame group, in which Prolame (1.25 μm) was added only 5 min before ischemia; I-R+Prolame+ODQ group, ODQ (15 μm) was added 15 min at the onset of the stabilization and before the addition of Prolame; I-R+Prolame+L-NAME group, L-NAME (50 μm) was added 15 min at the onset of stabilization and before the addition of Prolame. (B) Heart rate expressed as beats per minute. Values are expressed as mean ± SD of five independent heart preparations per group. The statistical test used was two-way ANOVA. ^∗^*P* < 0.001 I-R vs. I-R+Prolame and I-R+Prolame+ODQ; ^$^*P* < 0.05 I-R+Prolame vs. I-R+Prolame+ODQ; ^&^*P* < 0.001 I-R+Prolame vs. I-R+Prolame+L-NAME. (C) LVDP (left ventricular developed pressure). Values are expressed as mean ± SD of five independent heart preparations per group. The statistical test used was two-way ANOVA. ^∗^*P* < 0.001 I-R vs. I-R+Prolame and I-R+Prolame+ODQ; ^$^*P* < 0.05 I-R+Prolame vs. I-R+Prolame+ODQ; ^&^*P* < 0.001 I-R+Prolame vs. I-R+Prolame+L-NAME.

**Figure 3:**
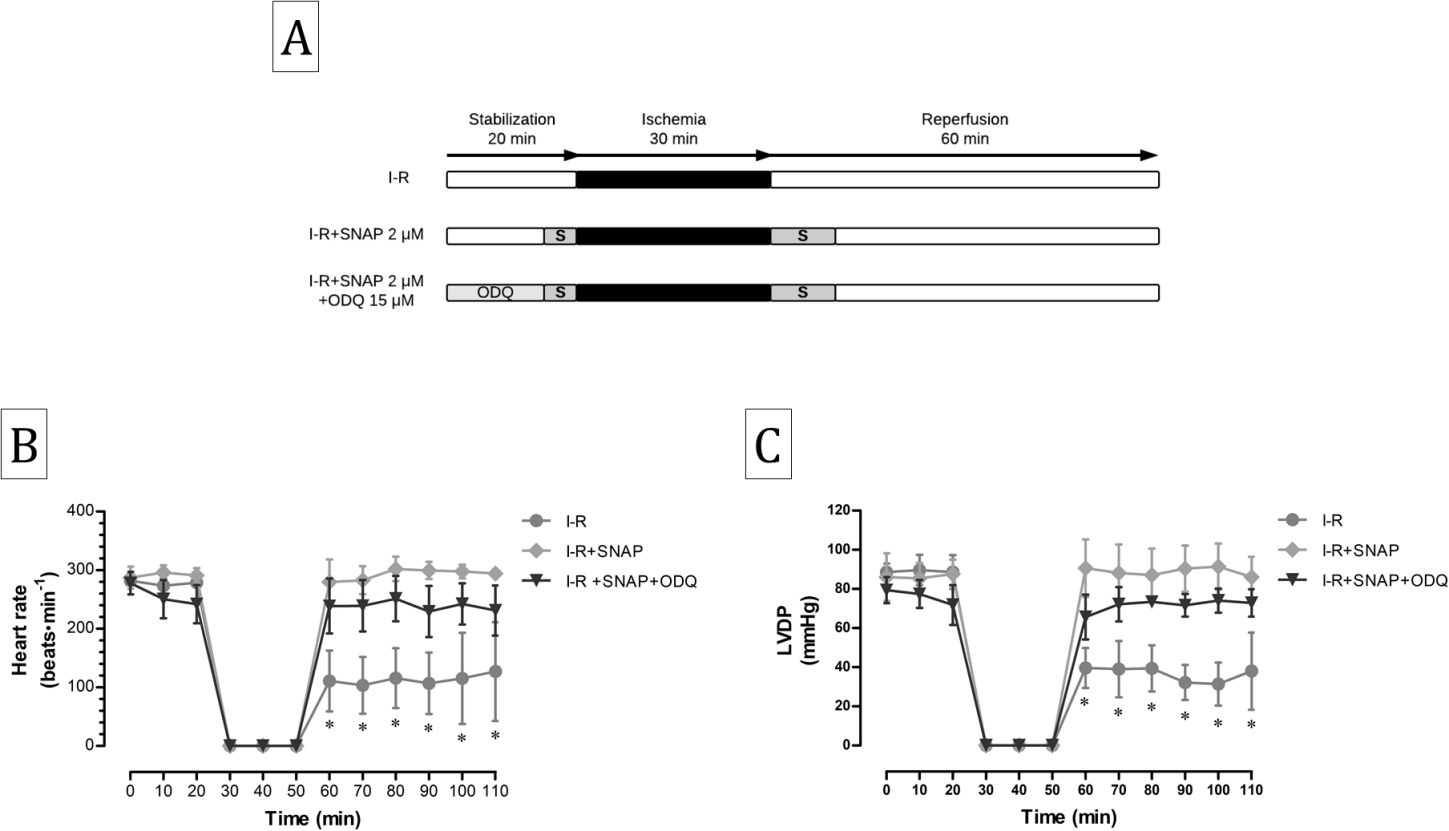
Effect of ODQ on the cardioprotection conferred by SNAP. (A) Schematic representation of the experimental procedure in the Langendorff model using the inhibitory compound ODQ. Ischemia-reperfusion (I-R) group, with 30 min of ischemia and 60 min of reperfusion; I-R+SNAP group, in which SNAP (two μm) was added 5 min before ischemia and 10 min at the start of reperfusion; I-R+SNAP+ODQ group, in which ODQ (15 μm) was added 15 min at the onset of stabilization and before the addition of SNAP (B). Heart rate expressed as beats per minute. Values are mean ± SD of five independent heart preparations per group. The statistical test used was two-way ANOVA. ^∗^*P* < 0.001 I-R vs. I-R+SNAP and I-R+SNAP+ODQ. (C) LVDP (left ventricular developed pressure). Values are expressed as mean ± SD of three independent heart preparations per group. The statistical test used was two-way ANOVA. ^∗^*P* < 0.001 vs. I-R+SNAP and I-R+SNAP+ODQ.

To evaluate the participation of the cGMP-dependent and independent mechanisms activated by NO in cardiac function recovery exerted by Prolame and SNAP, we administrated the NO synthase inhibitor N5-[imino(nitroamino)methyl]-L-ornithine, methyl ester, monohydrochloride (L-NAME) and the guanylate cyclase inhibitor ODQ to the I-R+Prolame hearts (Fig. 2A); whereas only ODQ was administrated to the I-R+SNAP hearts (Fig. 3A).

Neither ODQ nor L-NAME had effect in HR (Figs. 2B and 3B) or in LVDP (Figs. 2C and 3C) during the stabilization period before Prolame or SNAP administration. L-NAME abolished the cardioprotective effect conferred by Prolame in both HR (Fig. 2B) and in LVDP at the end of reperfusion (Fig. 2C); whereas ODQ administration to Prolame treated hearts diminished significantly HR from 257.4 ± 21.5 to 198.4 ± 19.2 beats min^−1^ (*P* < 0.05) and LVDP from 72.6 ± 4.1 to 58.6 ± 3.3 mmHg (*P* < 0.05) indicating that cGMP-independent mechanisms or other pharmacological properties non-related with NO production contribute to cardioprotection by Prolame. Blocking the cGMP-dependent pathway in the I-R+SNAP group, gives further clarity to the mechanisms activated by NO that contribute to cardioprotection and supports the idea that NO sustains S-nitrosylation processes. In this group, ODQ diminished HR from 294.2 ± 10.7 to 231.2 ± 42.7 beats min^−1^ (*P* < 0.05, Fig. 3B), but had no effect on LVDP values, which remain comparable to those observed in the SNAP group (86 ± 10.4 vs. 72.8 ± 6.9 mmHg; Fig. 3C).

**Figure 4:**
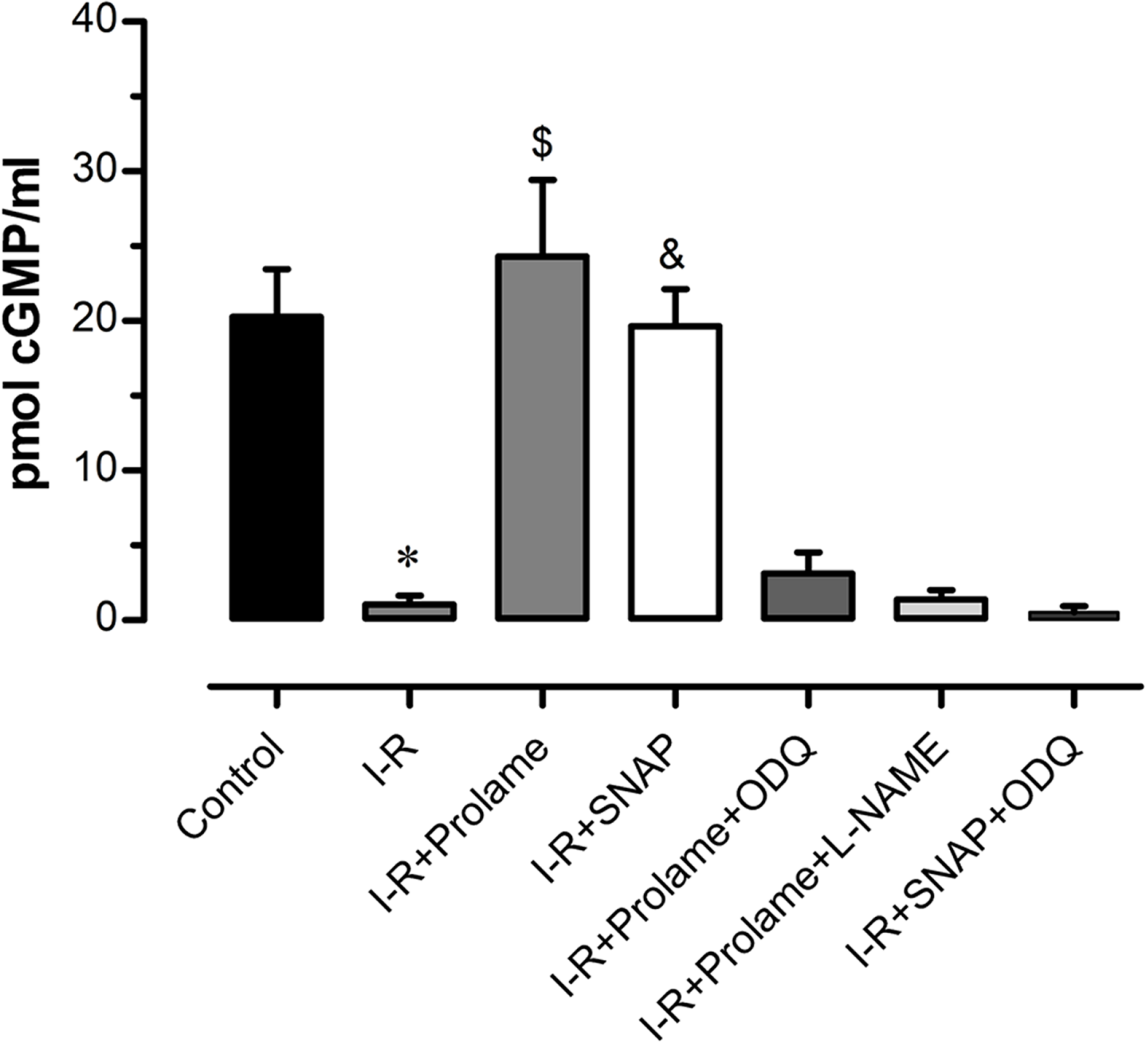
cGMP content. The levels of cGMP were measured at the end of the reperfusion in all the experimental groups. Values are expressed as mean ± SD of three independent heart preparations per group. The statistical test used was one-way ANOVA. ^∗^*P* < 0.05 I-R vs. Control, I-R+Prolame and I-R+SNAP; ^$^*P* < 0.05 I-R+Prolame vs. I-R+Prolame+ODQ, I-R+Prolame+L-NAME and I-R+SNAP+ODQ; ^&^*P* < 0.05 I-R+SNAP vs. I-R+Prolame+ODQ, I-R+Prolame+L-NAME and I-R+SNAP+ODQ.

To confirm that the inhibitors were efficiently acting on their corresponding targets, we measured cGMP content in all the experimental groups. The levels of cGMP decreased significantly in the I-R group in comparison with the Control group (1 ± 0.6 vs. 20.2 ± 3.2 pmol cGMP/ml; *P* < 0.05); whereas hearts treated with Prolame and with SNAP showed values comparable to those measured in the Control group (24.3 ± 5.1 and 19.6 ± 2.5 pmol cGMP/ml). Both ODQ and L-NAME diminished cGMP levels in the Prolame group to 3.1 ± 1.4 and 1.4 ± 0.6 pmol cGMP/ml respectively; whereas ODQ lowered cGMP to 0.5 ± 0.4 pmol/ml in SNAP-treated hearts (Fig. 4). These results unravel the participation of S-nitrosylation in the cardioprotection conferred by these compounds.

To discriminate between eNOS activation and other PI3K/Akt activated pathways in the conferred-cardioprotection, we also measured the levels of NO in the presence of ODQ and L-NAME. NO levels were 70.4 ± 2.8 and 24.8 ± 3.7 nmol nitrite/mg protein in the Prolame+ODQ and Prolame+L-NAME groups, respectively. L-NAME significantly decreased NO levels in comparison with the Prolame group, but such values were higher than those observed in the I-R group (3.03 nmol nitrite/mg protein, *P* < 0.05), indicating that the effects of Prolame are partially mediated through the NO. Also, differences were found between the I-R+SNAP+ODQ and the I-R+SNAP groups (86 ± 4.5 vs. 120 ± 7.3 nmol nitrite/mg protein).

### Effect of nitric oxide on apoptosis and necrosis

We performed TUNEL and DNA ladder analysis to evaluate the effect of NO on cell death regulation. Our results showed few apoptotic nuclei in I-R hearts, whereas the DNA ladder image clearly showed a mixture of necrotic/apoptotic cell death. Both Prolame and SNAP reduced condensed nuclei and DNA fragmentation (Figs. S1A–S1F).

### Effect of Prolame and SNAP on caspases and calpain-1 activities

**Figure 5:**
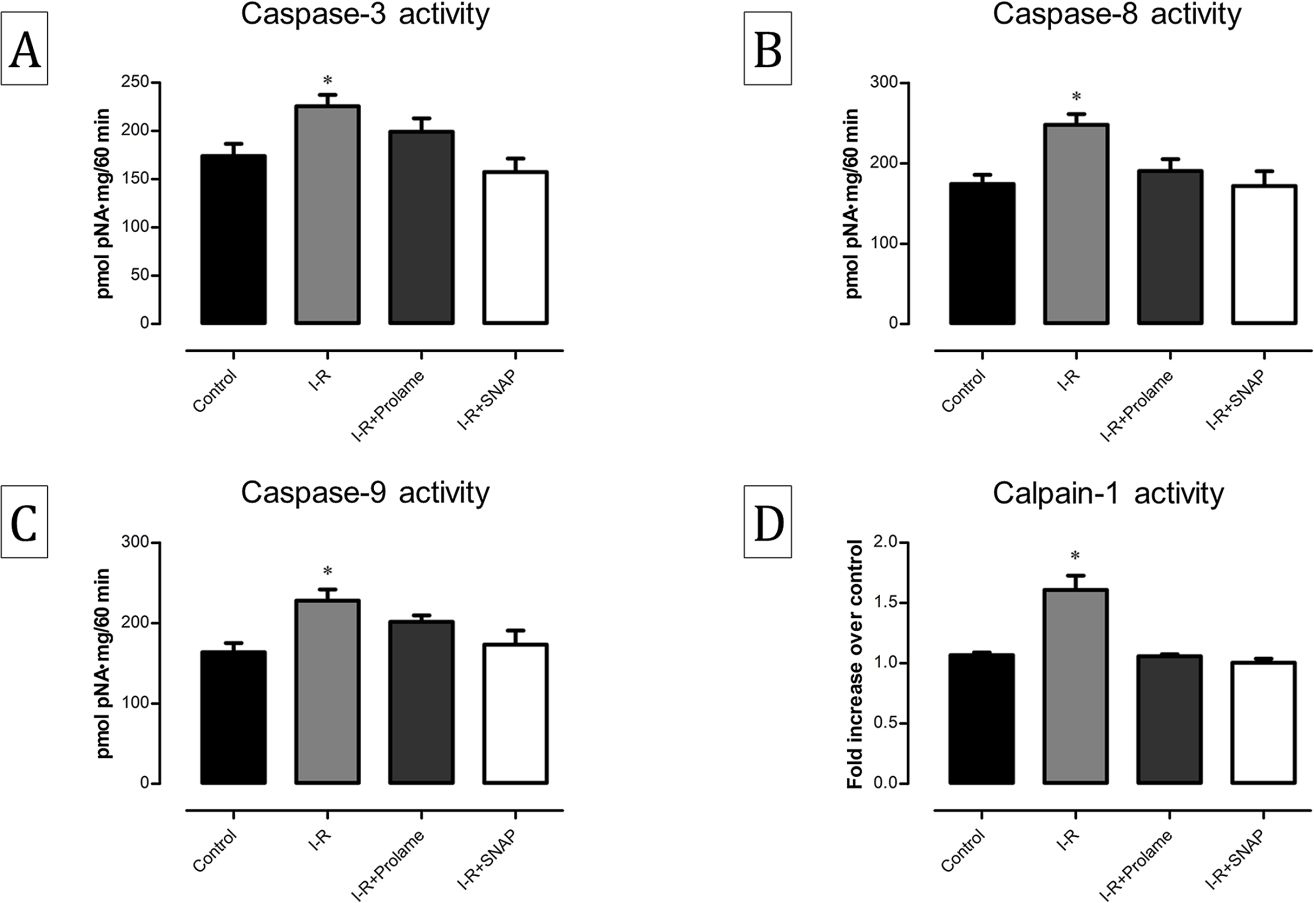
Activities of caspases-3, -8, -9 and calpain-1 in reperfused hearts treated with Prolame or SNAP. Activities of caspase-3 (A), -8 (B) and -9 (C) in all experimental groups. All caspase activities were measured as p-nitroaniline (pNA) release with respect to an aniline concentration curve. Data are expressed as mean ± SEM of six independent heart preparations per group. The statistical test used was one-way ANOVA. Caspase-3 activity: ^∗^*P* < 0.05 vs. I-R+SNAP; caspase-8 activity: ^∗^*P* < 0.05 vs. Control and I-R+SNAP; Caspase-9 activity: ^∗^*P* < 0.05 vs. Control and I-R+SNAP. (D) Activity of calpain-1 in all experimental groups. Data are expressed as mean ± SEM of at least nine independent heart preparations per group. The statistical test used was two-way ANOVA. ^∗^*P* < 0.05 vs. all groups.

We also evaluated the participation of apoptosis by measuring the activities of caspases using specific synthetic substrates. Caspase-8 and caspase-9 activities increased significantly in the I-R group as compared with the control group (*P* < 0.05) and SNAP diminished the activity of the three caspases (*P* < 0.05). Prolame slightly reduced their activity, but none statistical differences were found between I-R and I-R+Prolame hearts (Figs. 5A–5C). On the other hand, the activity of calpain-1 increased by 1.8-fold in I-R hearts as compared with the control group and diminished in both Prolame and SNAP-treated groups to basal levels (Fig. 5D). These results suggest that both pathways promote cell death during reperfusion, and that the activity of all the evaluated proteases is subjected to NO regulation.

We also measured the levels of cleaved caspase-3 (Fig. S2A) by western blot in heart homogenates. A significant increase of the p20 fragment of active caspase-3 was observed in I-R hearts which was diminished by SNAP or Prolame (*P* < 0.05). On the other hand, the levels of small subunit of calpain-1 does not showed significant differences between the experimental groups (Fig. S2B).

### Effect of Prolame and SNAP in SNO of caspase-3 and calpain-1

**Figure 6:**
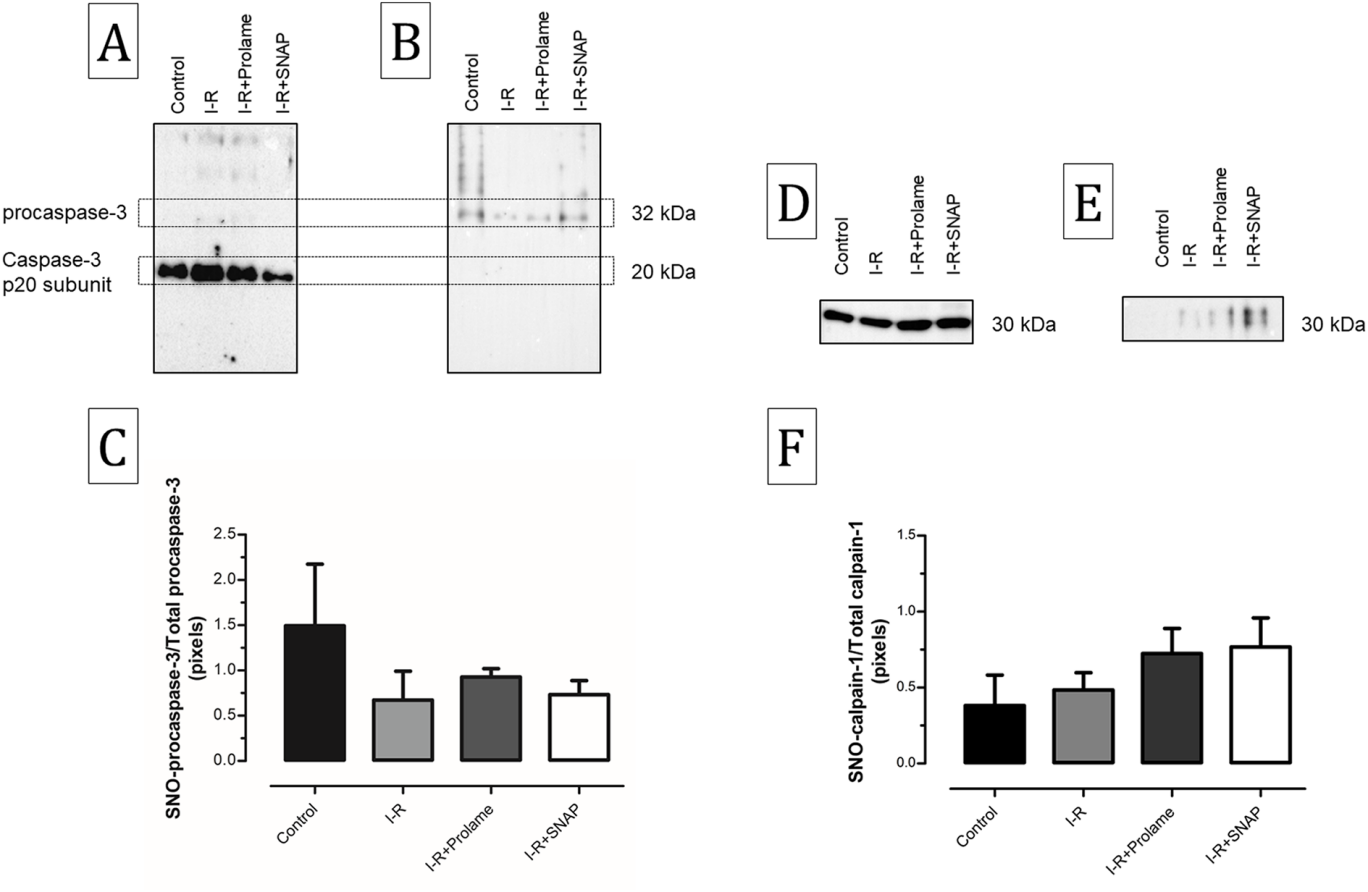
S-Nitrosylation of caspase-3 and calpain-1 in reperfused hearts treated with Prolame or SNAP. (A) Immunoblot of immunoprecipitated caspase-3 and (B) S-nitrosylated caspase-3. (C) bars representing the densitometric ratio between SNO procaspase-3/total procaspase-3. Data are mean ± SEM of three independent heart preparations per group. The statistical test used was one-way ANOVA. (D) Immunoblot of immunoprecipitated small subunit of calpain-1 and (E) S-nitrosylated small subunit of calpain-1. (F) bars representing the densitometric ratio between SNO calpain-1/total calpain-1. Data are mean ± SEM of five independent heart preparations per group. The statistical test used was one-way ANOVA.

It has been proposed that NO blocks apoptosis by modifying redox-sensitive cysteine residues in members of the caspase family. To determine if the observed inhibition in the activity of these proteases and of calpain-1 was associated with their levels of SNO, we labeled the S-nitrosylated residues of immunoprecipitated caspase-3 and calpain-1 with iodoacetyl TMT (iodo TMT™) reagent. Anti-caspase-3 antibodies pull down mainly the p-20 subunit of caspase-3 and in much lower extent the procaspase form. The amount of the cleaved subunit was lower in I-R+SNAP than in I-R hearts; conversely immunoprecipitated procaspase-3 increased in the SNAP group and diminished in I-R hearts. Anti-TMT antibodies only recognized the full-length caspase-3 and, although there are no significant differences, the S-nitrosylation signal tends to increase in the I-R+Prolame and I-R+SNAP groups in comparison with the I-R group (0.928 ± 0.09 and 0.733 ± 0.1 vs. 0.671 ± 0.3; respectively, Fig. 6A). On the other hand, the S-nitrosylation analysis of the small subunit of calpain-1 shows a tendency to increase in I-R hearts treated with Prolame or SNAP in comparison with the I-R group (0.724 ± 0.1 and 0.767 ± 0.2 vs. 0.485 ± 0.1, respectively, Fig. 6B). We recognize that direct S-nitrosylation experiments shown in Fig. 6 are not conclusive. Despite its widespread use, the biotin-switch technique is challenging and each step contains potential error sources. However, the maintenance of residual cardiac function in conditions were GMPc was depressed, have led us to maintain the speculation that S-nytrosylation plays a role in the cardioprotective effect of NO.

## Discussion

This study shows that both Prolame and SNAP diminish infarct size, maintain NO levels and prevent from cardiac reperfusion damage by activating cGMP-dependent and S-nitrosylation pathways. We observed that even though infarct size was negligible in reperfused hearts treated with Prolame, cardiac function diminished significantly in this group as compared with the Control group during reperfusion. Post-ischemic contractile dysfunction or “myocardial stunning,” associated with infarct size reduction has been described by other groups (Čarnická et al., 2011; Ford et al., 2001). Our results show that the NO donor SNAP is more efficient than Prolame to prevent from this benign form of reperfusion damage. Both compounds activate the cGMP-independent pathway in association with diminution in the activities of proteases linked to apoptosis and necrosis during reperfusion.

Necrosis is described as accidental non-apoptotic cell death that releases cellular content to the extracellular medium resulting in inflammation. On the other hand, apoptosis is a form of programmed cell death that is characterized by nuclear and cytoplasmic condensation and the formation of apoptotic bodies. It has been described that necrosis, apoptosis and in minor extent autophagy contribute to cell death in reperfused hearts; however, this issue remains under debate. While necrosis has been related with ischemic damage, apoptosis is thought to occur only during reperfusion and to coexist with necrotic death (Gottlieb et al., 1994; Vakeva et al., 1998; Zhao et al., 2000). The use of caspase inhibitors to reduce reperfusion damage have rendered conflicting results, weakening the idea of apoptosis relevance in reperfusion damage. For example, Chapman et al. (2002) reported that the selective caspase-3 inhibitor (S)-(+)-5-(1-(2-methoxymethylpyrrolidinyl)sulfonyl)isatin diminishes infarct size in isolated rabbit hearts and reduce apoptotic cell death; whereas other groups found that the same compound improves heart function independently of apoptosis regulation in rats (Kovacs et al., 2001; Ruetten et al., 2001).

Despite such controversial results, it has been reported that NO-activated pathways attenuate both apoptotic and necrotic cell death. The sGC-cGMP-PKG pathway modulates apoptotic cell death by increasing Bcl2/BAX ratio (Das, Xi & Kukreja, 2005) and reduces necrotic cell death through several mechanisms that ultimately maintain mitochondrial function. It is known that PKG phosphorylates L-type Ca^2+^ channels reducing calcium uptake during phase-2 of the action potential (Zhao, Greenstein & Winslow, 2017) and that activates the sarcoplasmic reticulum calcium ATPase (SERCA) promoting the dissociation of the regulator phospholamban after its phosphorylation at residue Ser^16^ (Inserte et al., 2014). It also induces the opening of mitoK_ATP_ channels (Penna et al., 2017), which enhance ROS production and promotes Protein Kinase C-epsilon (PKC-ε) activation (Costa & Garlid, 2008). The regulatory effect of this and other downstream kinases, for example, Akt, ERK1/2 and GSK3β (Hausenloy & Yellon, 2006) in conjunction with the attenuation of Ca^2+^ overload, inhibits the opening of the mitochondrial permeability transition pore (mPTP) avoiding irreversible cell injury (Costa & Garlid, 2008). Of note, even though PKG is considered the main intracellular mediator of cGMP, this cyclic nucleotide regulates other complex signaling cascades paramount to the cardiovascular system function, that includes several cGMP-protein kinases, cGMP-regulated phosphodiesterases (Das, Xi & Kukreja, 2008; Takimoto, 2012) and cyclic nucleotide-gated ion channels (Spinelli et al., 2018).

On the other hand, S-nitrosylation of cysteine residues of L-type channels (González et al., 2009) and of the ryanodine receptor (González et al., 2007) regulate their activity and maintain calcium homeostasis. Noteworthy, it has also been suggested that conforming and regulator proteins of the mPTP (adenine nucleotide translocase and cyclophilin D, respectively) have potential cysteine residues for S-nitrosylation (Chang et al., 2014).

The proposal that NO might block apoptosis by modifying redox-sensitive cysteine residues in the members of the caspase family, arise from observations that S-nitrosylation concurs with inhibition of caspase-3 activity in vitro and that antibodies against S-nitrocysteine immunoreact with this protease (Maejima et al., 2005). Our results show that the activity of this caspase, as well as of caspase-8 and caspase-9 decrease in the I-R+SNAP group. We found that S-nitrosocysteine post-translational modifications in caspase-3 were associated with the unprocessed enzyme and not with the active form, although this fragment was preferentially pulled down during immunoprecipitation. In this sense, Mannick et al. (1999) have proposed that during apoptosis, mitochondrial procaspase-3 is released into the cytoplasm where it is activated by denitrosylation. Also, it was reported that S-nitrosylation at cysteine 163 in the catalytic site of procaspase-3 inhibits its cleavage (Lai et al., 2011; Saligrama et al., 2014), whereas S-nitrosylated active caspase-3 (p17) has only been reported by a research group in immune cells infected by bacterial pathogens (Dunne et al., 2013). Regarding other caspases, studies in hepatocytes demonstrated that NO prevents the proteolytic activation of caspase-8 (Li et al., 1999) and, that S-nitrosylation of active caspase-8 decreases its activity and prevents death by apoptosis (Kim et al., 2000). Some reports indicate that the zymogen of caspase-9 is nitrosylated in mitochondria (Mannick et al., 2001) and that cysteine 325 is a critical S-nitrosylation site (Zhang et al., 2016).

We also found that the small subunit of calpain-1 showed increased S-nitrosylation in I-R+SNAP group as compared with I-R hearts. In this sense, a recent report showed that S-ntrosylation induced by S-nitroso-*N*-acetylpenicillamine (SNAP) concurs with diminution of cardiac calpain activity in murine cardiomyocytes subjected to hypoxia/reoxygenation, as well as in an in vivo reperfusion model (Totzeck et al., 2017). Calpain-1, a member of Ca^2+^-dependent cysteine proteases has a central role in myocardial injury during I-R, inducing cell death by apoptotic (Iwamoto et al., 1999; Gao & Dou, 2000) and by necrotic processes (Aguilar et al., 1996). It has been described that this protease mediates the translocation of the mitochondrial apoptosis inductor factor (AIF) to the cytosol and nucleus (Chen et al., 2011) and that also cleaves the pro-apoptotic protein bid, favoring the activation of the mitochondrial-dependent pathway of apoptosis (Chen et al., 2001). Other effects of cytosolic calpain activation are enzymatic degradation of proteins like actin, ankyrin, caldesmon, troponin T, troponin I, titin and desmone (Neuhof & Neuhof, 2014; Patterson et al., 2011; Potz et al., 2015), in addition to many other proteins, which produce membrane fragility and further calcium overload. This condition increases mitochondrial calcium concentration promoting the opening of the mPTP, which is considered the “hallmark event” in necrotic cell death. Thus, calpain-1 activation might induce necrosis by promoting calcium overload, but also as suggested by Thompson et al. (2016) it might directly modulate the mPTP. These group demonstrated that the administration of the calpain inhibitor MDL-28170 to isolated reperfused hearts decreases the release of lactate dehydrogenase and prevents from mPTP opening.

The factors that determine the preponderance of cGMP-dependent pathway over S-nitrosylation pathway remains to be clarified, but it has been speculated that the concentration and/or the site of NO generation might be relevant. In this sense, it has been reported that 100 μM of SNAP increases the levels of NO and cGMP in association with decreased contractility; whereas lower SNAP concentrations (0.1–1 μM) are related with lower NO content, increased contractility and S-nitrosylation in isolated rat hearts (González et al., 2008). In our experiments, although SNAP induces higher levels of NO than Prolame, cGMP levels were similar in both groups. ODQ abolished cGMP levels in association with partial loss of the cardioprotective effect, demonstrating the participation of the cGMP-independent pathway in hearts treated with Prolame and with SNAP. However, we observed that S-nitrosylation seems to be favored in the cardioprotection conferred by SNAP, as no differences in LVDP were detected between IR+SNAP vs. IR+SNAP+ODQ (Figs. 3B and 3C) in comparison with the same parameters in IR+Prolame vs. IR+Prolame+ODQ (Figs. 2B and 2C). We cannot fully attribute the cardioprotective effect of Prolame to the cGMP-dependent pathway, as this compound might exert additional effects to those related with NO signaling. The PI3K/Akt salvage pathway activate other downstream mediators besides eNOS, like the Glycogen Synthase Kinase 3 Beta, PKC-ε and the mitochondrial ATP-dependent potassium channel which regulates the opening of the mPTP (Ong et al., 2015).

In conclusion, we found that the cardioprotective effects of Prolame and SNAP are related with NO production and with diminution of caspases and calpain-1 activities. However, even that our results suggest the activation of S-nitrosylation in the cardioprotection conferred by NO to reperfused hearts, there was no a correlative link between the activity of the proteases and such process. We cannot discard that other proteins relevant to heart function might be modified by S-nitrosylation, neither dismiss the contribution of alternative mechanisms in the observed cardioprotection.

## Supplemental Information

10.7717/peerj.7348/supp-1 Supplemental Information 1 Heart performance, infarct size, nitric oxide content, cGMP content and proteases activities of reperfused rat hearts. Data indicate measurements obtained from least three independent heart preparations per group.

10.7717/peerj.7348/supp-2 Supplemental Information 2 TUNEL assay and DNA fragmentation analysis. Supplementary Figure 1. Representative images of *in situ* cell death assay in cardiac tissue from **(A)** Control, **(B)** I-R, **(C)** I-R+Prolame and **(D)** I-R+SNAP groups. Bar=50 μm. **E**. Bars in the graph show the amount of fluorescence of the nuclei in the 4 experimental groups. The statistical test used was 1-way ANOVA. ^*^P<0.05 vs. all groups. **F**. DNA fragmentation in the agarose gel shows a mixture of cell death by necrosis and apoptosis in the I-R samples.

10.7717/peerj.7348/supp-3 Supplemental Information 3 Content of caspase-3 and calpain-1 in reperfused hearts treated with Prolame or SNAP. **A**. Immunoblot of cleaved caspase-3 normalized with actin. Bars represent the densitometric ratio between p20 subunit/actin. Data are expressed as mean±SEM of three independent heart preparations per group. The statistical test used was 1-way ANOVA. ^*^P<0.05 vs. I-R+Prolame and I-R+SNAP. **B**. Immunoblot of the small subunit of calpain-1 normalized with GAPDH. The bars represent the densitometric ratio between small subunit calpain-1/GAPDH. Data are mean±SEM of three independent heart preparations per group. The statistical test used was 1-way ANOVA..

10.7717/peerj.7348/supp-4 Supplemental Information 4 Full blot of immunoprecipitated caspase-3. Lines 1 and 5, Control group; lines 2 and 6, I-R group; lines 3 and 7, I-R+Prolame group; lines 4 and 8, I-R+SNAP group; line 9 empty; line 10, molecular weights.

10.7717/peerj.7348/supp-5 Supplemental Information 5 Full blot of S-nitrosylated caspase-3. Lines 1 and 5, Control group; lines 2 and 6, I-R group; lines 3 and 7, I-R+Prolame group; lines 4 and 8, I-R+SNAP group; lines 9 and 10, empty; line 11, molecular weights.

10.7717/peerj.7348/supp-6 Supplemental Information 6 Full blot of immunoprecipitated calpain-1. Lines 1 and 5, Control group; lines 2 and 6, I-R group; lines 3 and 7, I-R+Prolame group; lines 4 and 8, I-R+SNAP group; lines 9, empty; line 10, molecular weights.

10.7717/peerj.7348/supp-7 Supplemental Information 7 Full blot of S-nitrosylated calpain-1. Lines 1 and 7, Control group; lines 2 and 8, I-R group; lines 3 and 9, I-R+Prolame group; lines 4 and 10, I-R+SNAP group; lines 5 and 6, empty; line 11, molecular weights.

## Additional Information and Declarations

### Data Availability

The following information was supplied regarding data availability: The raw measurements are available in Data S1.
