# Supplementary figures and images for: Cardioprotective effects of Prolame and SNAP are related with nitric oxide production and with diminution of caspases and calpain-1 activities in reperfused rat hearts
Source: PeerJ. 2019 Jul 29;7:e7348. doi: 10.7717/peerj.7348 (PMC6673759; doi:10.7717/peerj.7348)

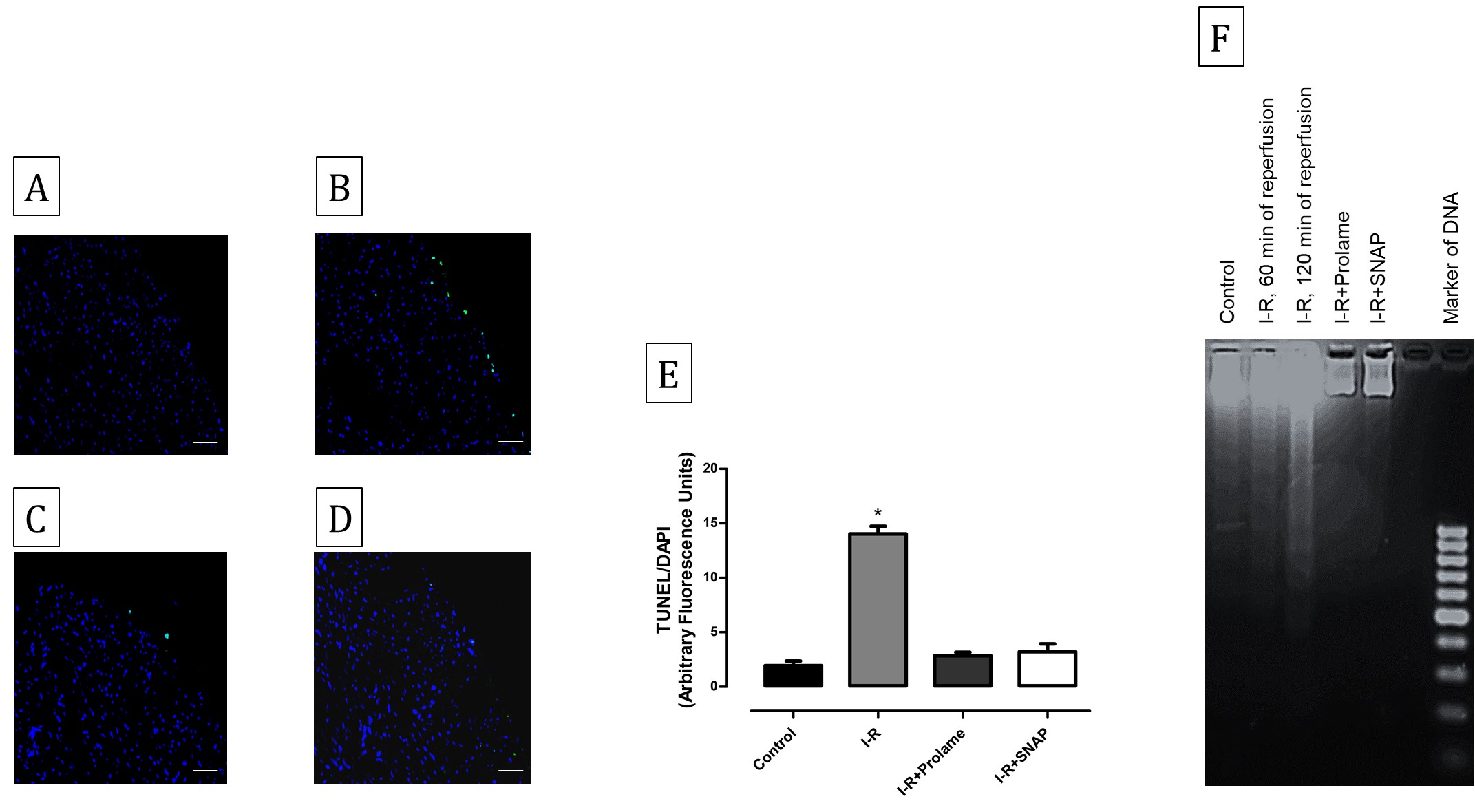

Supplement: Supplemental Information 2 — Supplementary Figure 1. Representative images of in situ cell death assay in cardiac tissue from (A) Control, (B) I-R, (C) I-R+Prolame and (D) I-R+SNAP groups. Bar=50 μm. E. Bars in the graph show the amount of fluorescence of the nuclei in the 4 experimental groups. The statistical test used was 1-way ANOVA. *P<0.05 vs. all groups. F. DNA fragmentation in the agarose gel shows a mixture of cell death by necrosis and apoptosis in the I-R samples. [file peerj-07-7348-s002.jpg]

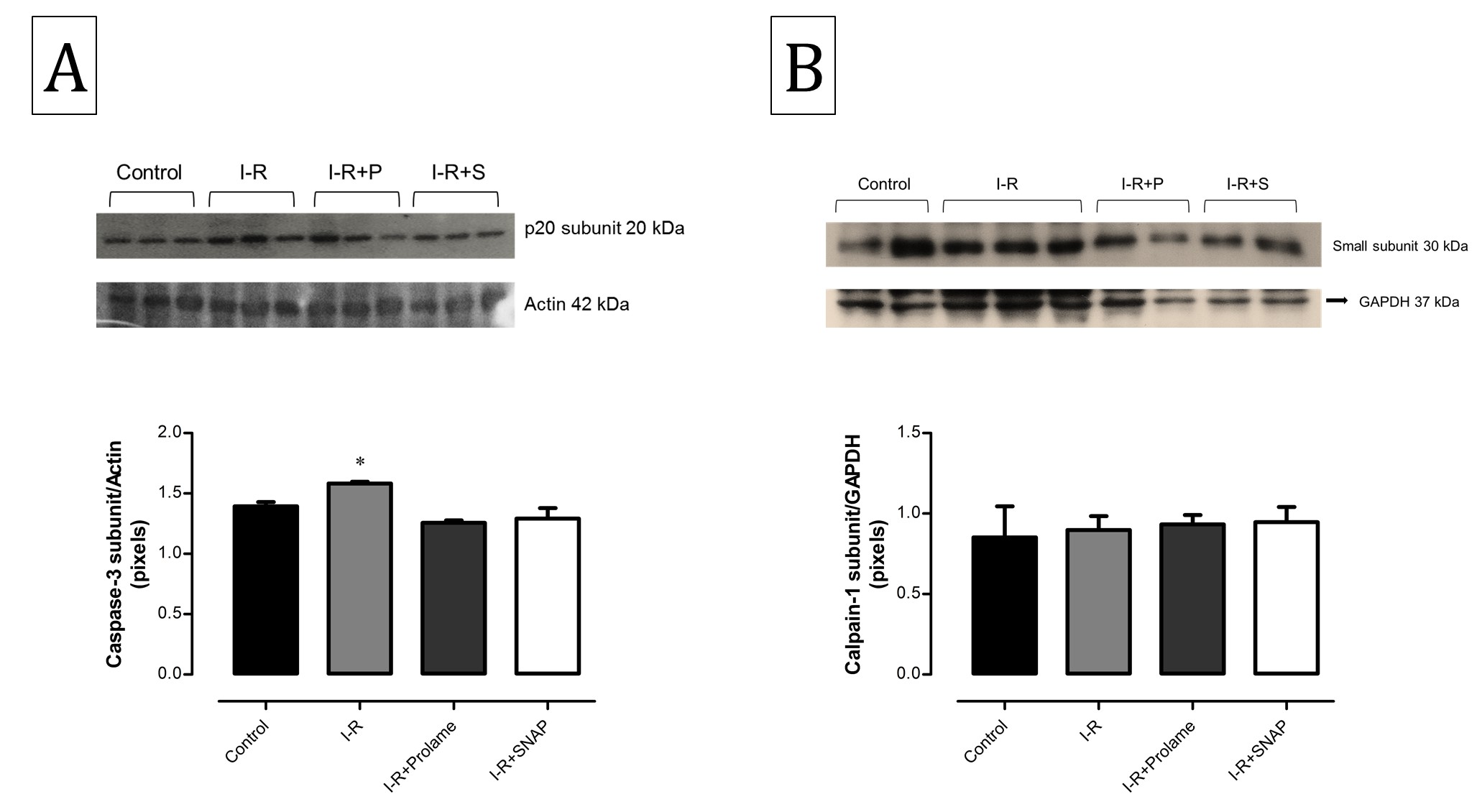

Supplement: Supplemental Information 3 — A. Immunoblot of cleaved caspase-3 normalized with actin. Bars represent the densitometric ratio between p20 subunit/actin. Data are expressed as mean±SEM of three independent heart preparations per group. The statistical test used was 1-way ANOVA. *P<0.05 vs. I-R+Prolame and I-R+SNAP. B. Immunoblot of the small subunit of calpain-1 normalized with GAPDH. The bars represent the densitometric ratio between small subunit calpain-1/GAPDH. Data are mean±SEM of three independent heart preparations per group. The statistical test used was 1-way ANOVA.. [file peerj-07-7348-s003.jpg]

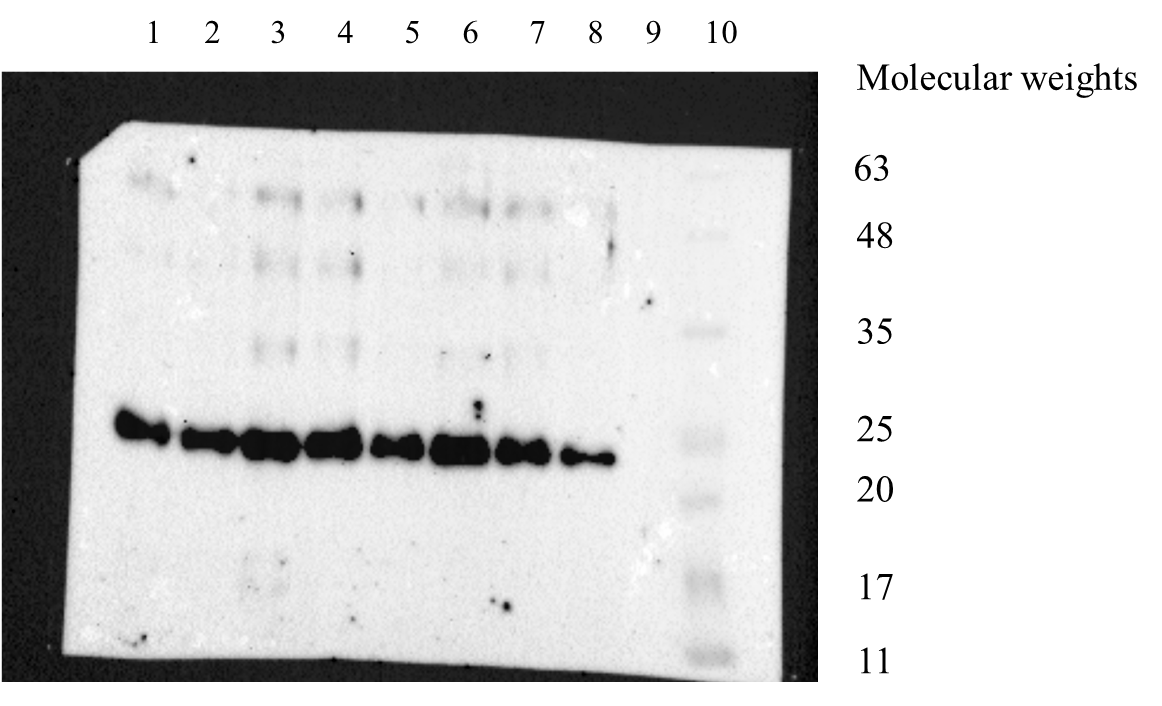

Supplement: Supplemental Information 4 — Lines 1 and 5, Control group; lines 2 and 6, I-R group; lines 3 and 7, I-R+Prolame group; lines 4 and 8, I-R+SNAP group; line 9 empty; line 10, molecular weights. [file peerj-07-7348-s004.png]

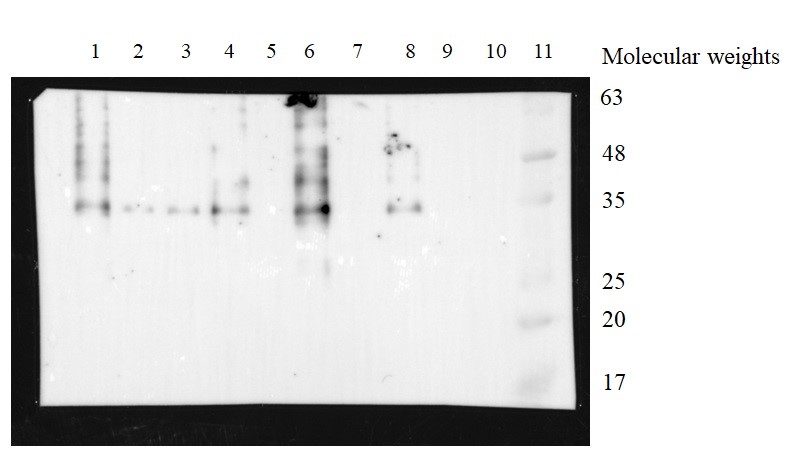

Supplement: Supplemental Information 5 — Lines 1 and 5, Control group; lines 2 and 6, I-R group; lines 3 and 7, I-R+Prolame group; lines 4 and 8, I-R+SNAP group; lines 9 and 10, empty; line 11, molecular weights. [file peerj-07-7348-s005.jpg]

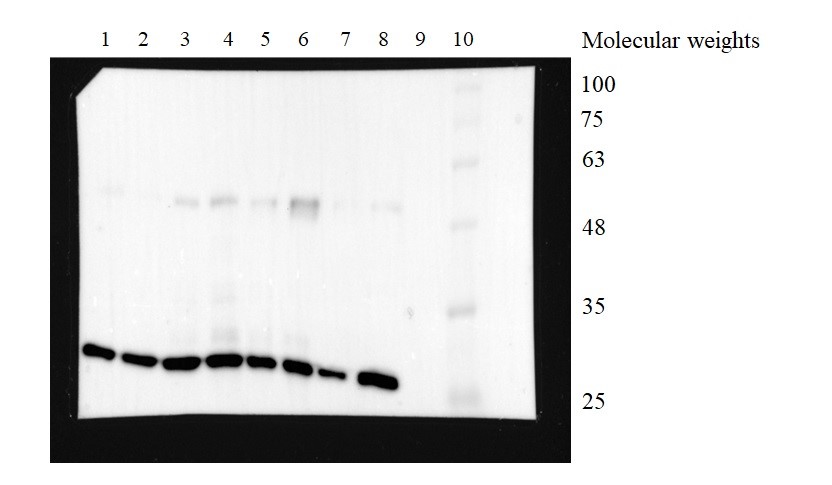

Supplement: Supplemental Information 6 — Lines 1 and 5, Control group; lines 2 and 6, I-R group; lines 3 and 7, I-R+Prolame group; lines 4 and 8, I-R+SNAP group; lines 9, empty; line 10, molecular weights. [file peerj-07-7348-s006.jpg]

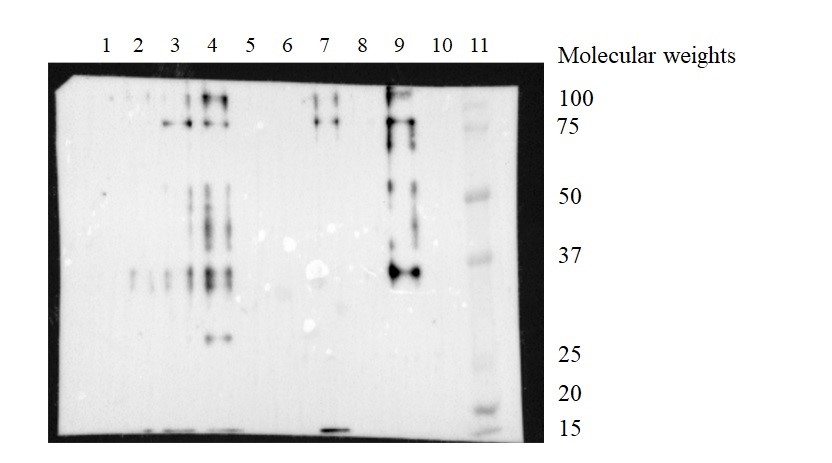

Supplement: Supplemental Information 7 — Lines 1 and 7, Control group; lines 2 and 8, I-R group; lines 3 and 9, I-R+Prolame group; lines 4 and 10, I-R+SNAP group; lines 5 and 6, empty; line 11, molecular weights. [file peerj-07-7348-s007.jpg]
